# The Admission of In-Patients under National Insurance

**Published:** 1911-12-16

**Authors:** J. Danvers Power

**Affiliations:** Thurloxton Manor, Taunton


### THE ADMISSION OF IN-PATIENTS UNDER NATIONAL INSURANCE.

*To the Editor of* THE HOSPITAL.

SIR,—In answer to your letter, I beg to say that I think the general tendency of recent taxation and legislation is to cripple all charity financially. But the estimates I have seen of the probable effect of the Insurance Bill, on the incomes of voluntary hospitals in particular, appear to me to go too far. The matter can only be one of conjecture at present; and, that being so, personally I do not see my way to build any definite argument on the assumption that a considerable number of beds will have to be closed in consequence of the Act.

As regards the eligibility of insured persons to "free hospital benefit," I think this depends on whether the benefit is *in-* or *out*-patient treatment.

People who can afford to pay a doctor, whether through a Friendly Society or privately, are not eligible for out-patient treatment now, unless the treatment is of a special character. They will be no more and no less eligible under the Act. But inasmuch as, under the Act, the presumption will be that a working man has a paid doctor of his own who ought to deal with the simple ailments and hurts, at present so extensively treated in casualty and out-patient departments, there ought, in my opinion, to be considerable relief in those departments of hospitals.

As far as in-patient treatment is concerned, I see no reason for doubting that every insured person will be just as much entitled to it as the sick-insurance members of Friendly Societies are now, and no more. When the case of a Friendly Society member is not suitable for home nursing, or obviously requires surgical skill beyond the scope of a general practitioner's work, he is not turned away from the hospital because he is insured; and I do not myself see how a change in the financial part of an insurance scheme affects that question. I was not aware that Mr. Lloyd George had said that insured persons "will have no right to come to the hospitals." But, in any event, whatever rights exist now are not, so far as I am aware, altered by any clause of the Insurance Bill. If they are altered hereafter by the action of the hospital governors, that is another matter.

I may add that, unless the fear of reduced hospital funds (and, by consequence, of closed beds) becomes a fact, I do not see why there should be any greater pressure on hospital accommodation after the Act than there was before. The Act cannot increase the volume of dangerous illness and serious accidents; and if people at present enjoy efficient hospital relief in these cases, as you mention, nothing but want of funds need prevent their enjoying it in the future.

But I certainly think that those who advocate State hospitals will be given adventitious opportunities of creating prejudice against the voluntary system by this Act. A great many improper cases, both for in- and out-treatment, will no doubt be referred to, and rightly refused by hospitals. Among these, mistakes by the hospitals themselves must sooner or later occur; and mistakes will be exploited by party politicians and have the worst interpretation put upon them in the usual way, regardless of truth or common-sense. I hope, therefore, it may be felt now, that the time has come for some sort of convention with the staffs of hospitals as to the proportionate number of request cases to be sent in by them, the number of vacant beds to be reserved for casualties, etc., and the system of filling up the remainder. Such an agreement, on reasonable lines, would, in my view, be the best way of preventing national insurance from becoming a menace to the voluntary system.—Your obedient servant, J. DANVERS POWER.

Thurloxton Manor, Taunton, December 4.

